# Characterization of Beak and Feather Disease Virus Genomes from Wild Musk Lorikeets (*Glossopsitta concinna*)

**DOI:** 10.1128/genomeA.01107-16

**Published:** 2016-10-06

**Authors:** Shubhagata Das, Sarker Subir, Katherine Adriaanse, Jade K. Forwood, Seyed A. Ghorashi, Shane R. Raidal

**Affiliations:** a School of Animal and Veterinary Sciences, Charles Sturt University, Wagga Wagga, New South Wales, Australia; b Department of Physiology, Anatomy and Microbiology, School of Life Sciences, La Trobe University, Melbourne, Victoria, Australia; c Healesville Sanctuary, Healesville, Victoria, Australia; d School of Biomedical Sciences, Charles Sturt University, Wagga Wagga, New South Wales, Australia

## Abstract

Three complete genomes of beak and feather disease virus (BFDV) were recovered from wild musk lorikeets (*Glossopsitta concinna*). The genomes consisted of 2,008 to 2,010 nucleotides (nt) and encode two major proteins transcribing in opposing directions. This is the first report of BFDV complete genome sequences obtained from this host species.

## GENOME ANNOUNCEMENT

Psittacine beak and feather disease (PBFD) is a chronic, debilitating, and ultimately fatal disease of *Psittaciformes* primarily involving the integument, alimentary, and immune systems of affected birds (1). The etiological agent of the disease, beak and feather disease virus (BFDV), is a compact circular, ambisense, single-stranded DNA (ssDNA) virus belonging to the genus *Circovirus* in the family *Circoviridae* (2, 3), and it is perhaps the simplest pathogen known to infect vertebrates. All *Psittaciformes* are considered susceptible to infection, since BFDV has been reported in more than 60 species of cockatoos and parrots (4–6). Lories and lorikeets are members of the relatively young (10 Ma) parrot subfamily *Loriinae*, which is chiefly confined to Australasia (7, 8), and new research has revealed that BFDV in lorikeets are sympatrically sequestered and divergent from those circulating in other psittacine hosts (9). Here, we characterize the BFDV genome from three wild Australian musk lorikeets (*Glossopsitta concinna*), a novel host species from the *Loriinae* subfamily.

Samples (blood and feather) from each of the wild-caught clinically suspected musk lorikeets were collected from distant locations in Australia. The first sample (identification [ID]: WHCC09-350) was collected in 2009 from Camden, New South Wales, Australia (34.0544°S, 150.6958°E), the second sample (ID: CS15-0142) was collected from Ballarat, Victoria, Australia (37.5622°S, 143.8503°E), and the third sample (ID: KAT-150065) was collected from Healesville, Victoria (37.6561°S, 145.5139°E). Genomic DNA was extracted from both blood and feather samples according to established protocols (6, 10, 11), and the whole-genome sequence was amplified using the primers and PCR conditions developed in our previous studies (9, 12, 13).

The first two complete genomes (GenBank accession numbers KM887917 and KX449319) of BFDV from the musk lorikeets consist of 2,008 nucleotides (nt), with G+C contents of 53.78% and 54.20%, respectively, while the third one (GenBank accession no. KX449322) has 2,009 nt in its genome and 54.20% G+C content. All three had genome structures similar to other BFDV genomes, which includes two major bidirectional transcribed open reading frames encoding a replication-initiator protein (Rep) and capsid protein (Cap). The newly amplified BFDV genomes shared 94 to 96% pairwise nucleotide identity with each other, but preliminary BLASTn analysis of the consensus of these sequences shows much greater sequence homology with BFDV genomes obtained from rainbow lorikeet (GenBank accession no. KM887935.1) and scaly breasted lorikeet (GenBank accession no. KM887946.1) (9). In addition, among the three newly assembled whole genomes, the Cap*-*encoding open reading frame 2 (ORF2) demonstrated more diversity (nucleotide identity 90 to 93%) than Rep*-*encoding ORF1 (nucleotide identity 95 to 98%). However, a phylogenetic analysis demonstrated that all these newly assembled genomes belong to the recently identified Lorinii clade of the BFDV phylogenetic tree (9), with strong consensus support.

This study highlights evidence of BFDV infection for the first time in musk lorikeets, as well as the genomic diversity of the virus, which may provide novel insights into the coevolutionary process of the virus in this host species.

### Accession number(s).

The three complete genomes of BFDV have been deposited at DDBJ/ENA/GenBank under the accession numbers KM887917, KX449319, and KX449322.
